# Editorial: Cardiac electronic remodeling and susceptibility to arrhythmias: an introduction and brief historical overview

**DOI:** 10.3389/fphys.2015.00196

**Published:** 2015-07-06

**Authors:** George E. Billman, Carlos L. del Rio

**Affiliations:** ^1^Department of Physiology and Cell Biology, The Ohio State UniversityColumbus, OH, USA; ^2^QTest Labs LLCColumbus, OH, USA

**Keywords:** arrhythmias, cardiac, ventricular fibrillation, atrial fibrillation, gap junctions, electrotronic coupling, myocardial electrical properties, myocardial infarction

The effective management of cardiac arrhythmias, either of atrial or of ventricular origin, remains a major challenge. Sudden cardiac death due to ventricular tachyarrhythmias remains the leading cause of death in industrialized countries (Hayashi et al., 2015) while atrial fibrillation is the most common rhythm disorder, the prevalence of this arrhythmia is increasing and accounts for nearly one quarter of ischemic strokes in the elderly population (Chugh et al., 2015). Yet, despite the enormity of the problem, effective therapeutic interventions remain elusive. In fact, several initially promising antiarrhythmic agents were found to increase rather than decrease mortality in patients recovering from myocardial infarction (Behr and Roden, 2013). The question then is what went wrong, why have these pharmacological interventions proven to be so ineffective? An obvious answer that is the drugs were designed to attack the wrong therapeutic target. Clearly, targeting single ion channels (using either isolated ion channels or single myocyte preparations) has proven to be less than effective. What then is the appropriate target? It is well established that cardiac electrical properties can vary substantially between single cells and intact preparations. One obvious example is the observation that action potential duration is much longer in isolated cells as compared to multi-cellular preparations or intact hearts. Due to the low electrical resistance between adjacent myocytes, the cells act in coordinated fashion producing an “electrotonic interdependence” between neighboring cells that results in a more uniform (and shorter) action potential duration than would be recorded in single uncoupled cells.

Engelmann (1875) was perhaps the first to recognize that the cardiac myocytes work as coordinated unit. He proposed what he called a “healing over” principle (as referenced by Janse, 2003) to describe how myocytes were joined to work and to live together (forming what is now known as a functional syncytium). However, the electrophysiological basis for the cell-to-cell-coupling was not established until the 1950's when Dr. Silvio Weidmann laid the “engineering” foundations of modern cardiac electrophysiology. His work provided the quantitative basis to Engelmann's “healing over” concept (Niggli et al., 2006), recognizing the importance of passive myocardial electrical properties to cardiac excitation/conduction (Weidmann, 1952). Weidmann (1952) applied earlier theoretical principles (Hodgkin and Rushton, 1946) to model a cardiac (Purkinje) fiber as a core-conductor/cable, having “a well-conducting protoplasm and by a thin surface membrane having a high resistance (*r_m_*) and a large capacity (*c_m_*) per unit area.” This “cable model” considers that the intracellular and extracellular potentials vary along the longitudinal axis only, and that both the cytoplasm and the extracellular spaces can be approximated as ideal ohmic conductors (with *r_i_* and *r_e_* respective resistances per unit length). Hence, propagating cardiac action potentials along a fiber can be described by the following second-order partial differential equation (PDE):

(1) $$\left(\frac{1}{r_{e}+r_{i}}\right)\frac{\partial^{2}}{\partial x^{2}}V_{m}=c_{m}\cdot \frac{\partial }{\partial t}V_{m}+I_{ionic}\left(V,t\right)$$

where *I_ionic_* is the nonlinear membrane ionic current density (μA/cm^2^), defined by the active/stochastic electrical properties of the cell. Alternatively, multiplying by *r_m_*, Equation (1) can be re-written as

(2) $${\text{λ}}^{2}\frac{\partial^{2}}{\partial x^{2}}V_{m}=\text{τ}\frac{\partial }{\partial t}V_{m}+V_{ionic}\left(V,t\right)$$

where $\text{λ}=\sqrt{\frac{r_{m}}{r_{e}+r_{i}}}$ is the length (and/or space) constant, a parameter that indicates how far a stationary current will “electrotonically” influence the voltage along the fiber, and τ = *r_m_c_m_* is the trans-membrane time-constant (e.g., Plonsey, 1969; Aidley, 1971).

Using this theoretical framework, Weidmann (1952) demonstrated that the electrical length/space constant was much larger than the cell length (defining the basis for the “electrotonic” modulation/homogenization of potentials across adjacent cells). He found that the internal longitudinal resistance (myoplasm in series with cell-to-cell contact) was much smaller than the membrane resistance. This result suggested the existence of low-resistance connections between neighboring cells, which Weidmann later demonstrated by studying and modeling (via the use of analog electrical circuits) the diffusion of potassium (Weidmann, 1960, 1966), showing that permeability of the intercalated disk to this ion was far greater than of the cell membrane. Subsequent investigations have identified the structural components (i. e., conforming proteins) of these inter-cellular junctional connections/channels, or “gap junctions” and have also confirmed their role in electrotonic coupling of the adjacent cells and in action potential propagation (for reviews see De Groot and Coronel, 2004; Wit and Peters, 2012; Dhein et al., 2014; Kleber and Saffitz, 2014). Alterations in these passive electrical properties can also lead to the generation of abnormal cardiac rhythms (both atrial and ventricular arrhythmias).

Myocardial infarction and/or acute ischemia provoke profound changes in the passive electrical properties of cardiac muscle (De Groot and Coronel, 2004). In particular, electrotonic uncoupling the myocytes disrupts the coordinated activation and repolarization of cardiac tissue. The resulting compensatory changes in ionic currents decrease cardiac electrical stability increasing the risk for life-threatening changes in the cardiac rhythm. Thus, the electrical properties of myocardial cells must be considered as a unit rather than in isolation. It is the purpose of this monograph to evaluate the largely neglected relationship between changes in passive electrical properties of cardiac muscle and arrhythmia formation. The book contains both state-of-the art reviews of the literature and original research articles that address various aspects of the effects of the passive electrical properties of the myocardium on cardiac rhythm. A brief summary of each chapter follow.

The role that changes in intrinsic properties of pacemaker cells (Yaniv et al., 2015), sinoatrial fibrosis (Csepe et al., 2015) and source sink (source—electrical charge for impulse generation; sink—the charge necessary to excite the surrounding tissue, impulse conduction) balance (Unudurthi et al., 2014) play in sinoatrial function and dysfunction are reviewed in chapters 2, 3 and 4, respectively. In a similar manner, the predominant role that gap junctions play in both normal and pathological changes in cardiac rhythm is reviewed in chapters 5–8. For example, Dhein et al. (2014) and Kleber and Saffitz (2014) review how electrotonic interactions, mediated both by junctional coupling proteins and geometrical/physiological factors, modulate source-sink phenomena in order to trigger/sustain normal cardiac rhythm and arrhythmogenesis (chapters 5 and 6). Kessler et al. (2014) further evaluate the contribution of heterogeneous gap junction remodeling to an increased risk for arrhythmias in several pathological conditions including hypertrophic, dilated, ischemic, and arrhythmic cardiomyopathies (chapter 7). Smit and Coronel (2014) next examine whether stem cells (implanted for stem cell replacement therapy) form functional electrotonic connections with cardiomyocytes and then evaluate the proarrhythmic risk that could result as a consequence of these connections (chapter 8). Trayanova et al. (2014) provide a state of the art assessment of computational modeling of atrial and ventricular arrhythmogenesis that result from disease induced changes in myocardial passive electrical properties (chapter 9), while Cabo (2014) uses similar computational approaches to analyze the effect on the dynamics of impulse propagation induced by simulated premature ventricular contractions in the infracted myocardium (structural heterogeneities caused by changes in gap junction conductance) (chapter 10). The functional significance of myofibroblast sodium currents on supraventricular arrhythmia formation is similarly investigated by Koivumäki et al. (2014) (chapter 11), while Walton et al. (2013) evaluate electrotonic modulation of repolarization using optical mapping techniques in species with large (pig) and small (rat) hearts (chapter 12). Despite the unequivocal mechanistic relationship(s) between passive electrical changes and arrhythmias, no study to date has directly assessed the ability of indices reflective of electrotonic coupling to stratify arrhythmic susceptibility *in vivo*. Therefore, del Rio et al. (2015) studied the effects of exercise-induced autonomic neural activation on electrotonic coupling as measured by myocardial electrical impedance in dogs know to be either susceptible or resistant to ischemically-induced ventricular fibrillation. They report that beta-adrenergic receptor activation enhances electrotonic coupling to a greater extent in susceptible as compared to dogs resistant to malignant arrhythmias and could thereby mask pro-arrhythmic repolarization abnormalities, an observation that may help explain false negative findings associated with exercise-stress testing in the clinic (chapter13).

The authors hope that this monograph will provide a better appreciation of the crucial role that myocardial passive electrical properties play in not only the maintenance of a normal cardiac rhythm but also how changes in these parameters can trigger atrial and ventricular arrhythmias. The application of this knowledge should facilitate the development of more effective anti-arrhythmic therapies.

## Conflict of interest statement

The authors declare that the research was conducted in the absence of any commercial or financial relationships that could be construed as a potential conflict of interest.
